# Dysregulated Homeostasis of Acetylcholine Levels in Immune Cells of RR-Multiple Sclerosis Patients

**DOI:** 10.3390/ijms17122009

**Published:** 2016-11-30

**Authors:** Maria Di Bari, Marcella Reale, Marta Di Nicola, Viviana Orlando, Sabrina Galizia, Italo Porfilio, Erica Costantini, Chiara D’Angelo, Serena Ruggieri, Stefano Biagioni, Claudio Gasperini, Ada Maria Tata

**Affiliations:** 1 Department of Biology and Biotechnologies Charles Darwin, Research, Center of Neurobiology Daniel Bovet, Sapienza University of Rome, 00185 Rome, Italy; maria.dibari@uniroma1.it (M.D.B.); viviana.orlando@uniroma1.it (V.O.); galiziasabrina1@gmail.com (S.G.); stefano.biagioni@uniroma1.it (S.B.); 2 Department of Medical, Oral and Biotechnological Science, University “G. d’Annunzio” Chieti-Pescara, 66100 Chieti, Italy; mreale@unich.it (M.R.); marta.dinicola@unich.it (M.D.N.); erica.costantini@unich.it (E.C.); chiara.dangelo@unich.it (C.D.); 3 School of Hygiene and Preventive Medicine, University “G. d’Annunzio” Chieti-Pescara, 66100 Chieti, Italy; porfilioitalo@gmail.com; 4 Department of Neurology and Psichiatry, Sapienza University of Rome, 00185 Rome, Italy; serena.ruggieri@gmail.com; 5 Department of Neurosciences, San Camillo Forlanini Hospital, 00185 Rome, Italy; c.gasperini@libero.it

**Keywords:** acetylcholine, acetylcholinesterase, butyrylcholinesterase, multiple sclerosis, cytokines

## Abstract

Multiple sclerosis (MS) is characterized by pro-inflammatory cytokine production. Acetylcholine (ACh) contributes to the modulation of central and peripheral inflammation. We studied the homeostasis of the cholinergic system in relation to cytokine levels in immune cells and sera of relapsing remitting-MS (RR-MS) patients. We demonstrated that lower ACh levels in serum of RR-MS patients were inversely correlated with the increased activity of the hydrolyzing enzymes acetylcholinesterase (AChE) and butyrylcholinesterase (BuChE). Interestingly, the expression of the ACh biosynthetic enzyme and the protein carriers involved in non-vesicular ACh release were found overexpressed in peripheral blood mononuclear cells of MS patients. The inflammatory state of the MS patients was confirmed by increased levels of TNFα, IL-12/IL-23p40, IL-18. The lower circulating ACh levels in sera of MS patients are dependent on the higher activity of cholinergic hydrolyzing enzymes. The smaller ratio of ACh to TNFα, IL-12/IL-23p40 and IL-18 in MS patients, with respect to healthy donors (HD), is indicative of an inflammatory environment probably related to the alteration of cholinergic system homeostasis.

## 1. Introduction

Multiple sclerosis (MS) is a chronic inflammatory disease of the central nervous system (CNS) characterized by the presence of sclerotic plaques that impair correct neuronal signal transmission [1]. Although the aetiology is still unclear, it is widely accepted that MS is caused by T cell auto-reactivity. In fact, these cells recognize some myelin like-proteins as “non-self”, causing the spread of an inflammatory response in the brain and spinal cord, leading to the destruction of myelin sheets.

Cytokines, the major regulators of the immune system, play a key role in modulating the inflammatory cascade in MS. Previous studies in MS patients and experimental animal models have highlighted that MS is mediated by Th1-type immune responses. IL-12 and IL-23 promote the development of Th1-type immune responses affecting the synthesis of several other cytokines including TNFα and IFNγ. Increased expression of IL-12p40 detected in acute MS plaques confirmed the pathogenic role for IL-12 in MS [2]. Together with IL-12, IL-18 acts as a link between innate and adaptive immune responses and participates in the pathogenesis of MS [3].

Acetylcholine (ACh), a classical neurotransmitter of the nervous system, can act as an immunomodulatory signal [4]. In immune cells, ACh is synthesized by the choline acetyltransferase (ChAT) enzyme [5]. The steady-state equilibrium of ACh is due to the action of two degradative enzymes: AChE, a specific cholinesterase that hydrolyzes selectively ACh, and BuChE, a non-selective cholinesterase that is able to hydrolyze ACh, in particular when co-expressed in ChAT-positive neurons localized in selected areas of the spinal cord and medulla oblongata [6]. Interestingly, studies in AChE-knockout mice have also indicated that BuChE is able to compensate for the AChE absence in ACh hydrolysis [7]. AChE is most abundant in the nervous system where ACh is rapidly hydrolyzed at the cholinergic synapses. BuChE is largely expressed both in the nervous system and in other tissue including blood, and its altered levels correlate with different neurological diseases [8]. The ACh storage mechanism is not yet clear, as the lymphocytes do not express vesicular acetylcholine transporter (VAChT) [9]. So it is likely that the ACh is synthesized and probably released through a non-vesicular release mechanism. In fact the expression of mediatophore and organic cationic membrane transporter novel type 1 (OCTN-1) appear to be involved in non-vesicular acetylcholine release in different tissues including the immune cells [10,11,12].

Recent reports have demonstrated a direct link between inflammation and the cholinergic system. In fact, through “neuronal” and “non-neuronal cholinergic anti-inflammatory pathways”, mediated by the vagus nerve and immune cells respectively, ACh actively participates in the modulation of cytokine synthesis and release [13,14,15]. The cholinergic muscarinic and nicotinic receptors mediate ACh functions in immune cells modulating the release of pro- and anti-inflammatory cytokines, respectively [11].

Considering this immuno-regulatory effect of ACh, it appears interesting to evaluate the role of the cholinergic system in MS. Previously we demonstrated that ACh levels in sera and cerebrospinal fluid (CSF) of RR-MS patients was lower than in HD [16]. Moreover, alterations of cholinesterase activities have been described in the nervous system, in sera and in plasma of MS patients [17,18]. Albeit cholinergic alterations being described in MS, a complete analysis of all cholinergic system members in the immune cells of MS patients, as well as an analysis demonstrating the co-presence of ACh and its hydrolyzing enzymes, is, at least, lacking. Similarly, the co-presence of peripheral cytokine levels and cholinergic markers in MS patients remains poorly investigated. In this context, we evaluated the expression and the activity of the cholinergic biosynthetic and degradative enzymes in sera and in peripheral blood mononuclear cells (PBMC) of RR-MS patients in relation to circulating ACh levels. The expression of two cholinergic membrane transporters involved in ACh release in biological fluids (i.e., mediatophore and OCTN-1) has been also evaluated. Finally the possible correlation of cholinergic marker expression and pro-inflammatory cytokine levels (i.e., IL-12/IL-23, IL-18 and TNFα) has been also estimated.

## 2. Results

### 2.1. ACh Levels and AChE and BuChE Activity in Sera of RR-MS Patients

**Figure 1:**
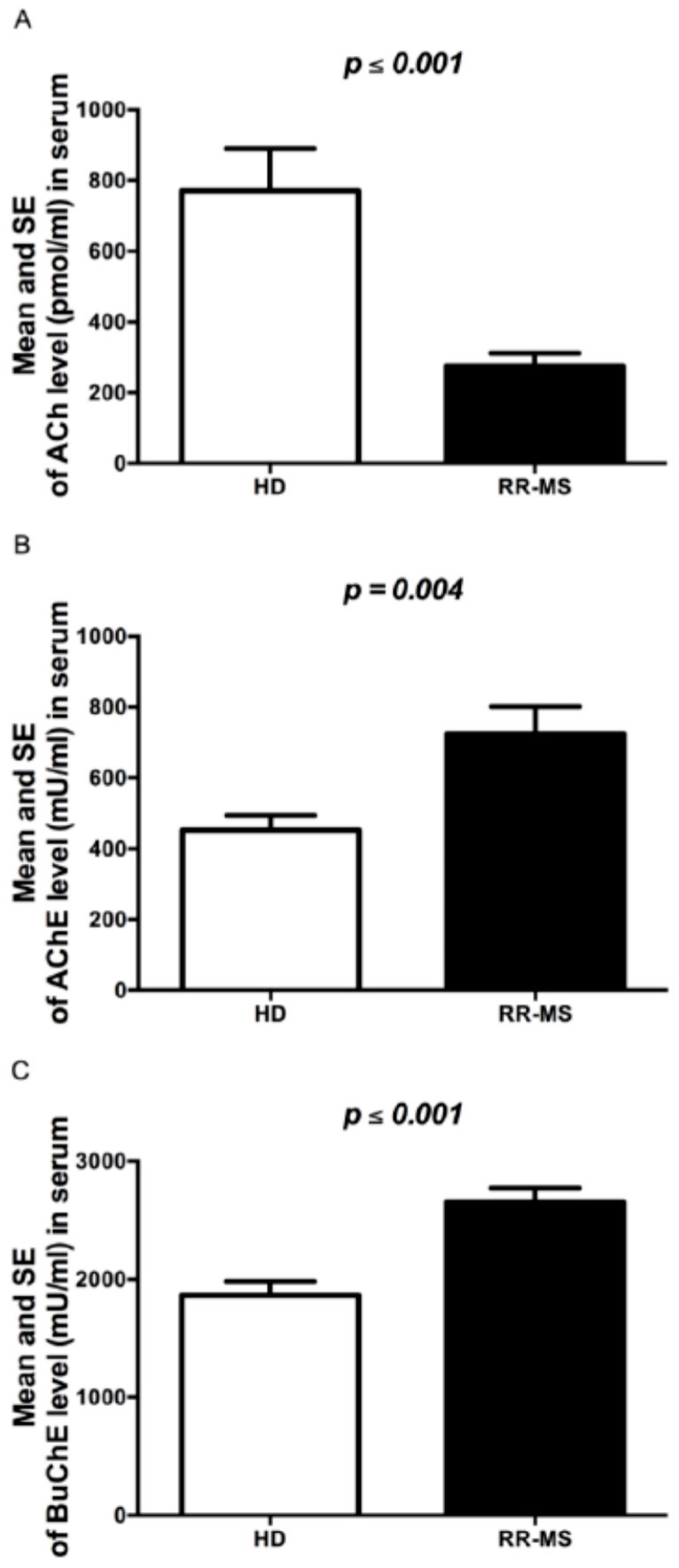
Cholinergic marker levels in serum of RR-MS patients and HD. (**A**) ACh levels (pmol/mL); (**B**) AChE activity (mU/mL); (**C**) BuChE activity (mU/mL). The bars represent the standard error (*p*-values reported in figure are relative to comparison between groups).

In this study we have confirmed the results obtained in our previous study on a small number of subjects [16]. As reported in Figure 1A, the ACh levels measured in the serum of the new extended group of 87 RR-MS patients were significantly lower, about four times, than in 60 HD. In order to evaluate whether the lower levels of ACh were dependent on a defect in ACh synthesis and/or hydrolysis, first of all, in the same RR-MS patients and HD subjects we evaluated the activity of the ACh hydrolyzing enzymes AChE and BuChE. The enzymatic activity resulted in significantly higher levels for both enzymes in sera of MS patients as compared to HD (Figure 1B,C). A negative correlation, although not statistically significant, between levels of ACh and AChE and BuChE activity was observed (Table 1, Figure 1).

### 2.2. Cholinergic Marker Expression in PBMC of RR-MS Patients

**Figure 2:**
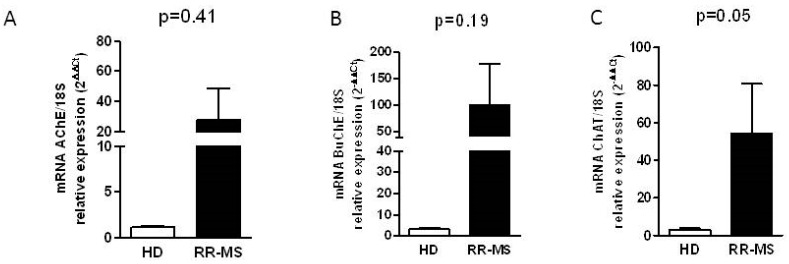
Analysis by qRT-PCR of cholinergic marker expression in PBMC of RR-MS and HD. Expression of AChE (**A**); BuChE (**B**) and ChAT transcript (**C**) levels. Further, 18S was used as housekeeping gene. The bars represent the standard error (*p*-values reported in figure are relative to comparison between groups).

**Figure 3:**
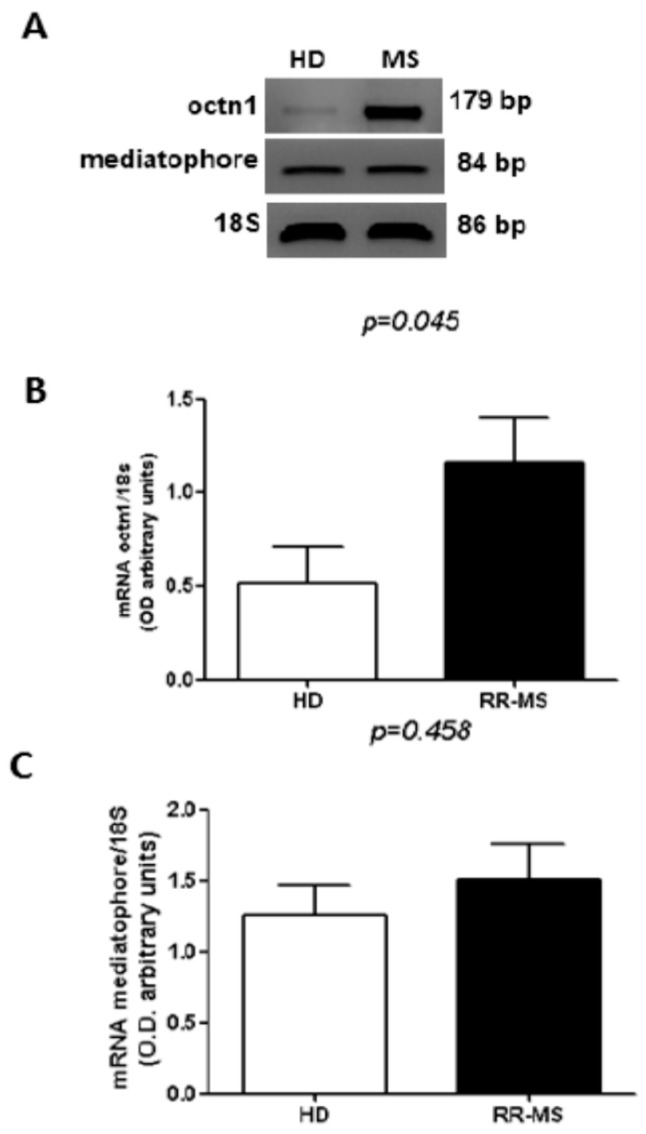
Expression of cholinergic markers in PBMC of RR-MS and HD. (**A**) A representative RT-PCR analysis of mediatophore and OCTN-1 performed in MS patients and HD; (**B**) densitometric analysis of the bands of OCTN-1 expressed in RR-MS and HD (*n* = 15); (**C**) densitometric analysis of the bands of mediatophore expressed in RR-MS and HD (*n* = 15). The bars represent the standard error (*p*-values reported in figure are relative to comparison between groups).

In order to evaluate whether the enhancement of cholinesterase activity depends on the increased expression of these enzymes, we analyzed the expression of AChE and BuChE transcripts in total RNA extracted by PBMC in a group of RR-MS patients (*n* = 15) and HD (*n* = 15) using qRT-PCR. Although we observed a high inter-individual variability, especially in the RR-MS group, the increase of the transcript levels of both hydrolyzing enzymes is evident, in particular for BuChE (Figure 2A,B). To verify if the ACh-synthesizing enzyme may upload the steady-state equilibrium of ACh levels, balancing the actions of higher cholinesterases, in RR-MS patients and HD subjects we evaluated the expression of the transcript for the ACh biosynthetic enzyme choline acetyltransferase (ChAT). As reported in Figure 2C, the levels of ChAT mRNA were higher in MS patients compared with HD subjects. Moreover, the analysis of the transcript for OCTN-1 and mediatophore, the two proteins responsible for the non-vesicular ACh release, typically expressed in immune cells [10,11], indicated higher OCTN-1 expression in PBMC of MS patients (Figure 3A,B), while the expression of mediatophore did not appear significantly changed (Figure 3A,C).

### 2.3. Expression of IL-12/IL-23p40, IL-18 and TNFα

**Figure 4:**
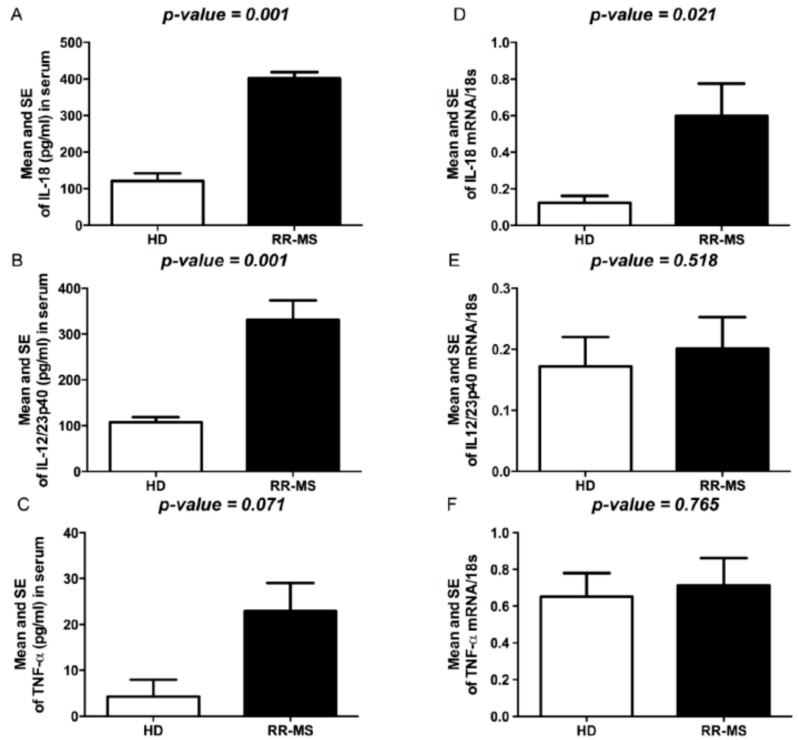
Cytokine levels in serum of RR-MS and HD. (**A**) IL-18 levels (pg/mL); (**B**) IL-12/IL-23p40 levels (pg/mL); (**C**) TNFα levels (pg/mL). Cytokine expression in PBMC of RR-MS and HD; (**D**) IL-18; (**E**) IL-12/IL-23p40; (**F**) TNFα 18S was used as housekeeping gene. The bars represent the standard error (*p*-values reported in figure are relative to comparison between groups).

We also evaluated serum levels of TNFα and of two cytokines involved in Th1 commitment such as IL-12p40, the common subunit shared by IL-12, IL-23 and IL-18. Significant differences in circulating levels of TNFα, IL-12/IL-23p40 and IL-18 were observed between RR-MS patients and HD (Figure 4A–C). To evaluate whether the serum cytokine levels could be related to production by PBMC, we also evaluated the spontaneous expression of selected cytokines in PBMC isolated from MS patients and HD subjects. Significantly higher mRNA levels of IL-18 were constitutively expressed in PBMC of MS patients with respect to HD subjects; IL-12/IL-23p40 and TNFα, although not significantly so, were higher in RR-MS than in HD (Figure 4D–F).

### 2.4. Relationship between Cholinergic Markers and Cytokine Levels

Since previous studies have hypothesized that ACh acts as a suppressor of inflammatory responses of lymphocytes [4], we have investigated the interrelationship of ACh generating and degrading enzymes and pro-inflammatory cytokines in MS patients and HD subjects. We found that AChE levels increased by >60% and BuChE levels increased by >40%, resulting in an overall decrease in ACh of 65% in MS patients compared to HD. Thus, AChE and BuChE, present at high levels in MS patients, alter the steady-state equilibrium of ACh and the ratio of AChE and BuChE to ACh was higher in MS patients than in HD (AChE/ACh 3.6 vs. 0.7, *p* < 0.001, and BuChE/ACh 13.0 vs. 3.1, *p* < 0.001). Although a non-significant association between ACh-hydrolyzing enzymes and IL-18, IL-12/IL-23p40 and TNFα levels was found, the smaller ratio of ACh to cytokines in RR-MS patients compared to HD (*p* < 0.001) is indicative of an inflammatory environment (Table 2).

## 3. Discussion

MS is a chronic inflammatory immune-mediated disease of the CNS [19] accompanied by severe peripheral and central inflammation responsible for myelin destruction and axon degeneration. The dysfunctions in the immune system in terms of antibodies and cytokine production are intimately related to the nervous system alterations; therefore, the control of the peripheral inflammation may contribute to ameliorate the central inflammation and to recover the nervous system functions [20].

The role of ACh in the pathogenesis of human diseases is emerging [21] and its involvement in cancer and various autoimmune pathologies has been, however, poorly investigated. Moreover, different nervous system disorders are characterized by cholinergic alterations that may be responsible for the increased inflammatory processes in the brain [22,23].

In fact, ACh is involved in the immune system functions regulating pro- and anti-inflammatory cytokine production and release (through muscarinic and nicotinic receptor activation, respectively) [24,25] and modulating B cells antibody production [26]. Current knowledge of cholinergic system activity in MS is quite limited. In experimental autoimmune encephalomyelitis mice (EAE), the main MS animal model, the use of AChE inhibitors causes a significant reduction of CNS lymphocyte infiltrates, confirming a role for ACh even in the modulation of central nervous system inflammation [15,27].

Our previous study has indicated that the ACh levels were significantly decreased in sera and CSF of RR-MS patients, suggesting a deficiency in the cholinergic system in MS [16]. In the present study, first of all, we have confirmed in a larger number of patients that ACh levels were significantly reduced in sera of RR-MS subjects, and, in order to better understand the involvement of ACh in the pathogenesis of MS, we have investigated the possible causes responsible for decreased ACh levels. To clarify if the possible cause of lower ACh levels was the increased hydrolysis or a decreased synthesis or release of ACh, we have analyzed the expression and the activity of the hydrolyzing enzymes AChE and BuChE. Interestingly the cholinesterase activity appeared significantly enhanced in sera of RR-MS patients compared to HD. This activity is dependent on the soluble form of AChE released in serum; in fact, the presence of the G4 form of AChE has been reported in human serum [28]. Although a complete characterization of the AChE molecular forms produced by hematopoietic cells is still lacking, it is known that all the immune cells (erythrocytes, platelets, lymphocytes and monocytes/macrophages) produce and release AChE in particular in stress conditions [29,30]. In the PBMC of the same MS patients, the activity is accompanied by an increase in AChE and BuChE transcripts. Several authors have hypothesized that variations in post-transcriptional mechanisms (i.e., microRNAs) regulating the levels of these cholinesterase may contribute to their altered expression [31].

In order to exclude that reduced ACh levels in serum may dependent also on deficiency in synthesis and release, we have evaluated the expression of the ACh biosynthetic enzyme, ChAT, and of the non-vesicular ACh transporters OCTN-1 and mediatophore [10,32] in RR-MS patients. The data obtained have indicated that both ChAT and OCTN-1 transcripts are significantly increased in RR-MS patients compared to HD, while mediatophore expression appears substantially unchanged. Our data demonstrate that all cholinergic components necessary for the synthesis and release of ACh are not defective in MS patients. However, we suggest that in MS patients, changes in the expression of AChE and BuChE would alter the availability of ACh and then this may lead to upregulation of ChAT and OCTN-1 expression in order to try to upload steady-state equilibrium of ACh levels. Further studies are needed to clarify if the increased transcript levels of ChAT and OCTN-1 were related to an up-regulation of the respective proteins. Altogether, our findings support the idea that the cholinergic dysfunction described in MS patients should be directly related to increased levels of ACh-degradative enzymes.

Th1-mediated responses are involved in the pathogenesis of MS and the Th1/Th2 paradigm has been useful in understanding the mechanism of immunomodulatory therapies. Thus, in our MS patients we have analyzed the levels of IL-18, a co-stimulant for Th1 cytokine production, IL-12, which plays a key role in Th1 differentiation, and TNFα production. In accordance with our previous study [16], we showed that the higher levels of pro-inflammatory cytokines IL-1β and IL-17 are related to decreased ACh levels, suggesting that ACh alteration may modulate IL-17 production or Th17 cell activation [16]. Interestingly, in the present study we found that in MS patients, increased BuChE and AChE activity matches with higher levels of IL-18, IL-12/IL-23p40 and TNFα. Our findings are consistent with studies on animal models for MS showing the amelioration of EAE after treatment with antibody against IL-12/IL-23p40 [33,34], and with the involvement of TNF in demyelinating disease [35]. The high serum levels of TNFα, IL-18 and IL-12/IL-23p40 are indicative of Th1 activation and an inflammatory environment. On the other hand, ACh levels allow us to hypothesize that the inability of RR-MS patients to promptly counteract the inflammation and the altered immune response should not be dependent either on a defect of ACh synthesis and release, or on the lack of activity and expression of nicotinic receptors. In fact, in our previous work we have demonstrated that the α-7 nicotinic receptors are expressed in PBMC of RR-MS patients and their stimulation can negatively modulate pro-inflammatory cytokines [36]. Our results, in agreement with previous observations indicating altered levels of cholinesterases in serum, plasma and CSF of MS patients [18], may explain why the use of cholinesterase inhibitors could decrease the release of pro-inflammatory cytokines, reduce neuro-inflammation, ameliorate EAE symptoms and improve cognitive deficit [37,38].

## 4. Materials and Methods

### 4.1. Subjects

Relapsing Remitting-MS patients were enrolled and followed at Department of Neuroscience of San Camillo-Forlanini Hospital (Rome, Italy). The diagnosis of RR-MS was confirmed according to revised Mc Donald Diagnostic Criteria [39] and RR-MS course was established by clinical parameters in agreement with recent classification [40]. Healthy donors (HD) were enrolled from the Transfusion Blood Bank Services of Chieti (Chieti, Italy) and were matched for age and gender. Mean age, mean disease duration and mean Expanded Disability Status Scale (EDSS) are shown in Table 3. The BBB impairment, refers to the ratio between CSF albumin/serum albumin, and represent an index of the functional alteration of the blood-brain barrier (BBB). All patients and HD signed an informed consent.

### 4.2. Blood Samples and PBMC Purification

Blood was collected in serum tubes (BD Biosciences, Oxford, UK) and centrifuged at 3000 rpm for 10 min. Aliquots of serum were stored at −20 °C. PBMC were separated from heparinized whole blood by density gradient centrifugation on Ficoll-Hypaque (GE Healthcare, Uppsala, Sweden) by the standard procedure.

### 4.3. Measurement of Acetylcholine Levels

ACh was measured by commercial colorimetric/fluorimetric kit (Abcam, Cambridge, UK), as previously reported [16]. The level of Ch/ACh (pmol/well) was calculated by plotting the fluorescence of each sample in relation to choline standard curve. The measurement of the fluorescence was obtained using Glomax Multi Detection System (Promega, Milano, Italy) at λ Ex/Em 535/587 nm.

### 4.4. Cholinesterase Activity Determination

Cholinesterase activities were measured in sera of RR-MS patients and HD by Ellman assay [41], using 1 mM final concentration of acetyl-thiocholine iodide as substrate. In order to evaluate the contribution of AChE and BuChE to the total cholinesterase activity, 1.4 × 10^−5^ M BW284c51 or 1.4 × 10^−5^ M lysivane were respectively added as appropriate inhibitors, in the reaction mixture containing 0.33 mM DTNB (di-nitro-thiocyanobenzene) in 0.1 M phosphate buffer, pH 7.

Enzyme activity was expressed as mU; 1 mU corresponding to 1 nmole of substrate hydrolyzed/min at 30 °C.

### 4.5. RNA Extraction, qRT-PCR and Semi-Quantitative RT-PCR Analysis

Total RNA was extracted from PBMC using Blood Total RNA purification kit (FMB, Italy) and then digested with DNAse I (Ambion-Life Technologies Italia, Monza, Italy). One microgram of RNA was reverse transcribed into cDNA according with the manufacturer’s instructions. Quantitative real-time PCR were performed with GoTaq qPCR Master mix (Promega, Milano, Italy) using Cycler IQTM Multicolor Real Time Detection System (Biorad, Hercules, CA, USA). Quantification of gene expression was calculated using the comparative threshold cycle (*C*_t_) method, normalized to the 18 S control and efficiency of the RT reaction (relative quantity, 2^−ΔΔ*C*t^). For semiquantitative RT-PCR (sqRT-PCR) the densitometric analysis of the bands were performed using ImageJ software (Bethesda, MA, USA) (OD amplicon/OD housekeeping gene). These values are expressed as arbitrary units. The sequences of the primers used in RT-PCR analysis are reported in Table 4.

### 4.6. Cytokine Measurements

Human cytokine levels in serum were quantified using ELISA assays for IL-18, IL12/IL-23p40 and TNFα. ELISA assay was conducted with commercial kits (R & D System, Minneapolis, MN, USA) according to the manufacturer’s instructions. The plates were read at 450 nm and the absorbance was transformed to pg/mL, using calibration curves prepared with cytokine standards. The intra- and inter-assay reproducibilities were >90%.

### 4.7. Statistical Analysis

The quantitative variables were summarized as mean, standard error (SEM) and interquartile range (IQR), qualitative variables as frequency and percentage. The results are reported separately for each of two groups (HD and RR-MS). Statistical analysis was performed using non-parametric tests when the distribution of the variables was not normal, as assessed by the Shapiro-Wilk test. Mann-Whitney U test was applied for assessing the comparison of the quantitative variables between two groups. Fisher’s exact test was applied for comparison of the qualitative variables. The correlations between serum ACh and other quantitative variables were estimated by non-parametric Spearman *rho* correlation coefficient (σ). All statistical tests were evaluated at an alpha level of 0.05. Statistical analysis was performed using SPSS^®^ Advanced Statistical 11.0 software (SPSS Inc., Chicago, IL, USA).

## 5. Conclusions

For the first time we demonstrate an inverse correlation between cholinergic hydrolyzing enzyme activity and ACh levels in serum of RR-MS patients. The present data suggest that the dysregulation of ACh levels may contribute to the pathogenesis of MS, contributing to maintaining the pro-inflammatory state. The reestablishment of the correct ACh homeostasis in the immune system of MS patients, with the consequent regulation of pro-inflammatory cytokines, may be a new, interesting therapeutic perspective for MS. Although, to date, there are not sufficient data in MS patients on the use of AChE inhibitors [42,43], it is known that EAE mice chronically treated with AChE inhibitors present amelioration of the disease symptoms and reduction of neuroinflammation and demyelination [27]. However, the possible activation of nicotinic receptors with selective agonists may represent a possible alternative to the therapeutic modulation of the immune system in MS, as suggested by our previous study, indicating the ability of nicotinic receptor agonists to decrease the pro-inflammatory cytokine production in PBMC of MS patients [36].

## Figures and Tables

**Table 1:** Spearman Rho correlation coefficient (relative *p*-value) of ACh levels vs. cholinergic markers.

Cholinergic Markers	RR-MS Group	HD Group
AChE	−0.121 (0.334)	−0.118 (0.411)
BuChE	−0.122 (0.327)	−0.112 (0.432)
OCTN-1	0.239 (0.323)	−0.135 (0.661)
Mediatophore	−0.059 (0.810)	−0.171 (0.576)
ChAT	−0.249 (0.319)	

**Table 2:** Mean and standard error of ratio between ACh/cholinergic hydrolyzing enzymes and ACh/cytokines.

Ratio	RR-MS Group	HD Group	*p*-Value ^a^
AChE/ACh	3.6 ± 0.3	0.7 ± 0.1	<0.001
BuChE/ACh	13.0 ± 0.4	3.1 ± 0.1	<0.001
ACh/TNFα	8.9 ± 1.8	140.6 ± 14.9	<0.001
ACh/IL-18	0.5 ± 1.9	5.0 ± 0.3	<0.001
ACh/IL-12/IL-23p40	0.6 ± 0.2	5.6 ± 0.5	<0.001
ACh/TNFα mRNA	291.1 ± 16.7	863.6 ± 15.8	<0.001
ACh/IL-18 mRNA	1007.5 ± 0.2	2038.0 ± 1.1	<0.001
ACh/IL-12/IL-23p4 mRNA	1019.0 ± 0.5	3022.5 ± 0.5	<0.001

^a^ Mann-Whitney U test.

**Table 3:** Characteristics of the subjects involved in this study.

Variable	HD Group (*n* = 60)	RR-MS Group (*n* = 87)	*p*-Value
Gender, *n* (%)			0.601 ^a^
Male	7 (11.7)	21 (16.7)	
Female	53 (88.3)	66 (83.3)	
Age (years), median (range)	41 (19–66)	38 (18–59)	0.377 ^b^
BBB impairment, median (range)	-	5.5 (2.8–15.2)	
Duration of disease (years), median (range)	-	5 (3–6)	
EDSS, median (range)	-	2.5 (0.0–6.0)	

BBB: blood-brain barrier; EDSS: Expanded Disability Status Scale; ^a^ Fisher’s exact test; ^b^ Mann-Whitney U test.

**Table 4:** Primers sequences used in qRT-PCR and semiquantitative RT-PCR analysis.

**Primers Sequences (qRT-PCR)**	**Forward**	**Reverse**
AChE	5′-TCTACGCCTACGTCTTTGAA-3′	5′-AACTCGATCTCGTAGCCGT-3′
BuChE	5′-TATGGGACTCCTTTGTCAGT-3′	5′-TGAAGCCAGGAGCACCATA-3′
ChAT	5′-AGAGCAGCAGGAAGCTGAT-3′	5′-TTTTCTGCCGAGGAGGCTAA-3′
IL-12/23	5′-CCGTTCACAAGCTCAAGTATG-3′	5′-GCAGAATGTCAGGGAGAAGTAG-3′
IL-18	5′- CAGTCAGCAAGGAATTGTCTC-3′	5′-GAGGAAGCGATCTGGAAGG-3′
TNFα	5′-CCTTCCTGATCGTGGCAG-3′	5′-GCTTGAGGGTTTGCTACAAC-3′
18 S	5′-CCAGTAAGTGCGGGTCATAAGC-3′	5′-AACGATCCAATCGGTAGTAGCG-3′
**Primers Sequences (sqRT-PCR)**	**Forward**	**Reverse**
OCTN-1	5′-CTGCCCAGGCGTTATATCAT-3′	5′-GGTTGGGTAGAGCTCAGCAG-3′
Mediatophore	5′-TCCTCTCCACAAAGTAGACCCT-3′	5′-TGTTCGTTCTGGAATGAGGA-3′
